# Thermo-Mechanical Coupling Model of Bond-Based Peridynamics for Quasi-Brittle Materials

**DOI:** 10.3390/ma15207401

**Published:** 2022-10-21

**Authors:** Haoran Zhang, Lisheng Liu, Xin Lai, Hai Mei, Xiang Liu

**Affiliations:** 1 Hubei Key Laboratory of Theory and Application of Advanced Materials Mechanics, Wuhan University of Technology, Wuhan 430070, China; 2 Department of Engineering Structure and Mechanics, Wuhan University of Technology, Wuhan 430070, China

**Keywords:** bond-based peridynamics theory, quasi-brittle materials, thermo-mechanical coupling, crack propagation

## Abstract

The mechanical properties of quasi-brittle materials, which are widely used in engineering applications, are often affected by the thermal condition of their service environment. Moreover, the materials appear brittle when subjected to tensile loading and show plastic characteristics under high pressure. These two phenomena manifest under different circumstances as completely different mechanical behaviors in the material. To accurately describe the mechanical response, the material behavior, and the failure mechanism of quasi-brittle materials with the thermo-mechanical coupling effect, the influence of the thermal condition is considered in calculating bond forces in the stretching and compression stages, based on a new bond-based Peridynamic (BB-PD) model. In this study, a novel bond-based Peridynamic, fully coupled, thermo-mechanical model is proposed for quasi-brittle materials, with a heat conduction component to account for the effect of the thermo-mechanical coupling. Numerical simulations are carried out to demonstrate the validity and capability of the proposed model. The results reveal that agreement could be found between our model and the experimental data, which show good reliability and promise in the proposed approach.

## 1. Introduction

Thanks to their unique brittle characteristics, materials such as ceramics, rocks, and concrete are widely used in various engineering fields, including the aerospace, armor protection, and construction industries. However, temperature, as an essential environmental factor, can impact the mechanical properties and service performance of the above materials during operation [1]. It may even cause serious accidents associated with the thermal cracking of surrounding rocks upon the underground storage of nuclear waste [2] and heat-induced spalling of building concrete [3]. Therefore, to ensure the safety and load-bearing capacity of quasi-brittle engineering materials, their mechanical properties and damage behavior must be understood under the thermos-mechanical coupling.

To study the behavior of quasi-brittle materials undergoing thermal damage, numerous experimental facilities have been developed and utilized, such as X-ray computed tomography (X-CT) [4], acoustic emission (AE) [5], scanning electron microscopy (SEM) [6], and thermal stress devices (TSD) [7]. These approaches have enabled researchers to significantly improve their knowledge about the hermos-mechanical properties of the materials. However, experimental studies are not only time-consuming and laborious, but it is also challenging to observe the sprouting and extension of micro-cracks inside the material in situ. Thus, by providing a detailed and cost-effective prediction, numerical methods shed insight into the mechanical failure processes in quasi-brittle materials.

Extensive numerical investigations have been devoted to understanding the thermal damage behavior of quasi-brittle materials, which are generally based on continuum mechanics approaches. Various numerical approaches have been developed on the basis of the Finite Element Method (FEM) [8,9,10], Extended Finite Element Method (X-FEM) [11], Finite Difference Method (FDM) [12], and Boundary Element Method (BEM), etc. Since the numerical approaches mentioned above are based on continuum mechanics, in which partial differential equations need to be solved to find the numerical solution, the ability to deal with the problem of cracks and fractures is often limited, even after the introduction of special-made shape functions in X-FEM. Moreover, special treatment is often needed to maintain the stability of the system, determine when the nucleation and crack happens, and keep track of the crack propagation path, such as the remeshing and level-set method. In this regard, those methods are highly dependent on numerical modeling and discretization.

Peridynamics (PD) theory [13], a nonlocal continuum theory proposed in recent years, is a theory in which spatial differential equations are replaced by integral equations, which provide a uniform framework for both continuities and discontinuities. Unlike the partial differential equations in classical theory, the controlling equations of PD still hold at geometric discontinuities, such as crack discontinuities, making it possible to model crack nucleation and expansion along arbitrary paths. Hence, PD can efficiently deal with fracture problems concerning brittle fractures in solids, complex fracture morphologies, crystal dislocations, and high-speed impacts of geological materials under explosive action.

Until recently, PD has been employed extensively to investigate the thermal impact damage mechanisms of brittle materials such as rocks, ceramics, and concrete. For instance, Chen et al. [14] proposed the refined thermo-mechanical, fully coupled PD approach by applying the PD differential operator to a classical thermal differential equation. This method is suitable for studying the heat conduction and thermal deformation of, and damage to, concrete structures. Shou and Zhou [15] added the thermal expansion coefficient of a solid material to the thermal coupling equation of the non-ordinary state-based Peridynamics (SB-PD). They first used the temperature field to calculate the deformation gradient tensor, then the latter was introduced into the non-ordinary SB-PD motion equation to realize the thermo-mechanical coupling process. Moreover, this method was subsequently employed to simulate the thermal cracking of rocks, and the simulation results showed good convergence with experimental results. Bazazzadeh et al. [16] developed a thermo-mechanical coupling PD model for simulating crack extension in ceramics using an adaptive mesh. Specifically, this model was adaptively transformed based on a stretching control criterion for mesh discretization and then applied in the desired finite region, thereby enabling the prediction of complex cracking forms. Yang et al. [17] proposed a new method of characterizing the mineralogical composition and distribution of heterogeneous rock materials using fully coupled conventional thermo-mechanical equations of PD. The model under this method has the ability of describing the thermal-force damage behavior of granite after thermal cycling treatment. Taking the study [18] as an example, Liu et al. [19] further increased the tangential bond force by considering the influence of the bond on the rotation effect so that the ceramic model developed by Chu et al. could break through the Poisson’s ratio limit and be adopted to more types of ceramic materials. Wang et al. [20] derived a microscopic thermal conductivity parameter that links various micro- and macro-geometric conditions based on a weakly coupled thermoelastic, non-ordinary, state-based Peridynamics (OSB-PD) model by analyzing the temperature distribution. Furthermore, they proposed a tensile damage criterion that takes into account the softening effect of stretched parts. However, the above study focuses only on the tensile damage of quasi-brittle materials, ignoring the nonlinear mechanical behavior caused by the generation of micro-cracks in the compression phase. As a result, the simulated results of quasi-brittle materials under thermal coupling deviated from the experimental data.

In this study, a model suitable for the failure of quasi-brittle materials under thermo-mechanical coupling is proposed, and the effect of temperature within the elastic and plastic stages is considered. To verify the reliability and validity of this model, numerical simulations of ceramics under a heating load and the pre-cracked Brazilian disk under uniaxial compression were conducted. Finally, two-dimensional granite plates were exposed to cold, uniaxial compression experiments after heat treatment were conducted, and the coincidence between the simulated and experimental results were analyzed.

## 2. Thermo-Mechanical Coupling Model

In this section, the classic fully coupled thermo-mechanical BB-PD model, in which the bonds remain elastically deformed during deformation until broken and are not applicable to quasi-brittle materials, is first introduced. Then, the mechanical behavior of quasi-brittle materials in tension and compression is presented. The Peridynamics model for quasi-brittle materials is presented in detail in Section 2.3, and the substance of this paper, i.e., the study of thermal effects in the tensile and compression phases, is presented. Finally, the numerical discretization and time integration methods of the proposed model are presented.

### 2.1. Fully Coupled Thermo-Mechanical Equation

**Figure 1:**
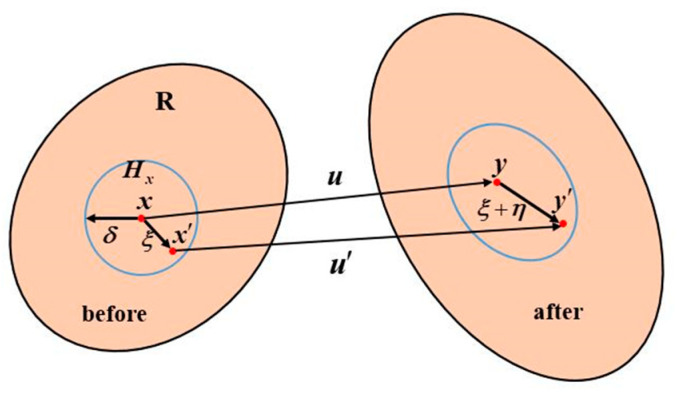
Schematic diagram of the BB-PD model.

Unlike the partial derivative of deformation with respect to the spatial coordinate in continuum mechanics, the BB-PD theory adopts the spatial integral equation, which can be applied to discontinuous bodies [21]. As shown in Figure 1, each material point x in a region **R** of an object interacts with all other points within its neighborhood radius $H_{x}$. For the domain $x^{\prime }$, the material point is called a neighborhood particle of the material point x. When a solid is deformed under an external load, the matter points x and $x^{\prime }$ arrive at the post-deformation positions y and $y^{\prime }$ through displacements u and $u^{\prime }$, respectively, and |ξ| is the distance between the two particles before the deformation and |ξ+η| is the distance between the two particles after the deformation.

In contrast to the classical derivation of the heat equation [22], PD laws are derived based on irreversible thermo-mechanicals, i.e., energy conservation and free energy density functions. The fully coupled thermo-mechanical equations for BB-PD are as follows [23,24,25]:

(1) $$\rho c_{v}\dot{T}\left(x,t\right)=\int_{H}\left(\kappa \frac{\tau }{\left|\xi \right|}-T_{0}\frac{c\alpha }{2}\dot{e}\right)dV^{\prime }+h_{s}\left(x,t\right)$$

(2) $$\rho \ddot{u}\left(x,t\right)=\int_{H}\frac{\xi +\eta }{\left|\xi +\eta \right|}csdV^{\prime }+b\left(x,t\right)$$

Here, Equation (1) is the PD thermal diffusion equation with a structural coupling term, where $c_{v}$ is the specific heat capacity, κ is the thermal conductivity of the bond in the PD system, $h_{s}\left(x,t\right)$ is the rate of heat production per unit volume at the point of matter x at time t. The temperature difference between the substance points x and $x^{\prime }$ can be expressed as $\tau =T\left(x,t\right)-T\left(x^{\prime },t\right)$, where $T_{0}(c\alpha /2)\dot{e}$ are the deformation terms caused by heating and cooling, $T_{0}$ is the reference temperature, and $\dot{e}$ is the rate of change of bond lengths, denoted as:

(3) $$\dot{e}=\frac{\xi +\eta }{\left|\xi +\eta \right|}\cdot \dot{\eta }$$

Equation (2) is the equation of motion for the BB-PD system with a temperature coupling term. In this equation, ρ is the mass density; $\ddot{u}\left(x,t\right)$ is the acceleration of the matter point x at time t; H is the neighborhood range of the matter point x; and $V^{\prime }$ is the volume of the matter point $x^{\prime }$; c is the bond constant; α is the coefficient of thermal expansion; b(x,t) is the force density of the matter point x at time t; s is the stretch rate between x and $x^{\prime }$; and $T_{avg}$ is the average temperature of the matter points x and $x^{\prime }$, respectively, expressed as:

(4) $$s=\frac{\left\|\xi +\eta \right\|-\left\|\xi \right\|}{\left\|\xi \right\|}-\Delta T_{avg}\cdot \alpha$$

(5) $$\Delta T_{avg}=\frac{\Delta T+\Delta T^{\prime }}{2}$$

### 2.2. The Characterization of the Mechanical Behavior of Quasi-Static Brittle Materials

For quasi-brittle materials such as ceramics and concrete, the damage models under compression and tension are distinct. Under tension, the behavior is brittle, while a more ductile behavior can be observed under compression. The brittle behavior of materials under tensile loading is attributed to macro-crack formation. On the other hand, the ductile behavior of ceramics under compression can be explained by micro-crack formation and plasticity [26,27,28].

**Figure 2:**
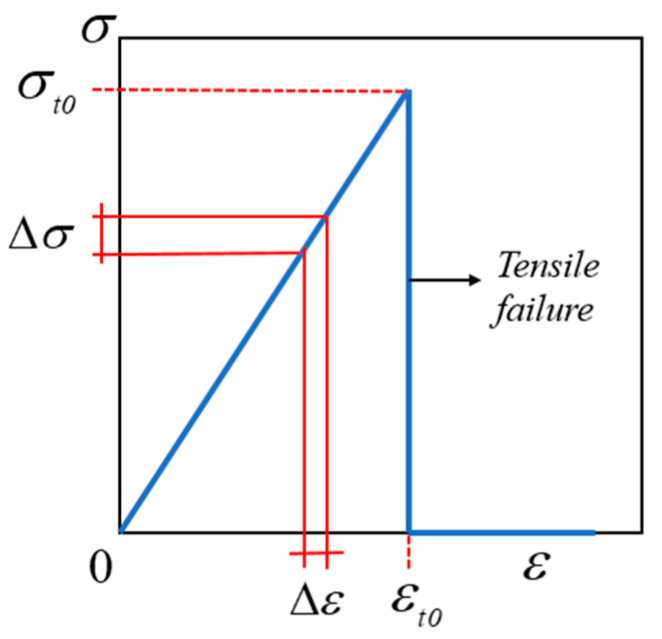
Stress–strain diagram of tensile behavior.

Under tensile loading, the quasi-brittle materials undergo direct, brittle damage at the end of elastic deformation, with essentially no plastic deformation. Figure 2 depicts a typical stress–strain diagram of the quasi-brittle material model in the tensile phase, showing a linear relationship between stress and strain, where the material breaks down and loses its load-bearing capacity after reaching the tensile-strength limit of the material.

When a quasi-brittle material is subjected to a compressive load, the model remains intact at the initial stage, and as the load continues to increase, structural crushing occurs internally due to the generation of micro-cracks and lattice plasticity, and the interaction of these cracks leads to a decrease in the compressive strength of the quasi-brittle material, a phenomenon described as plastic-softening behavior. The quasi-brittle material is treated as elastic material before damage occurs, and after damage occurs, it is treated as material that remains intact but whose strength decreases with the accumulation of damage.

**Figure 3:**
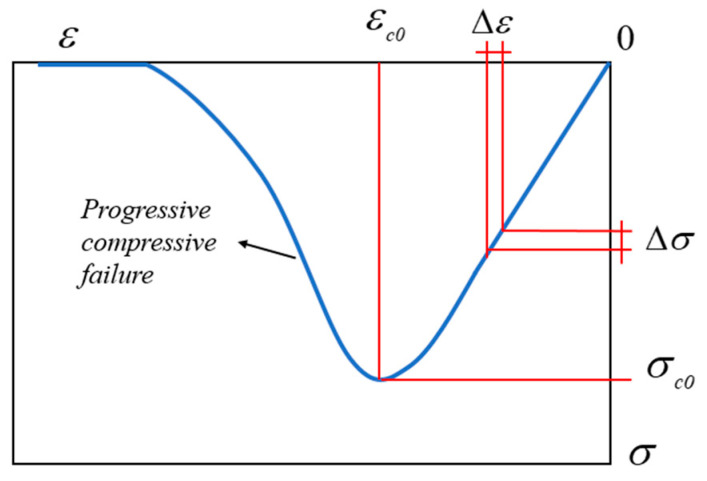
Stress-strain diagram of compression behavior.

Figure 3 shows a typical stress–strain diagram for the ideal quasi-brittle material which is typical of ultra-high-strength concrete [29]. Before reaching the elastic limit of the material, for a sufficiently small segment, the relation is close to linear. After exceeding the elastic limit, micro-cracks appear in the concrete, resulting in an inelastic response that differs from the plastic-flow behavior of ductile materials, as shown by the fact that the strength of the material decreases with the accumulation of plastic damage, i.e., the material begins to soften. Finally, the concrete is completely broken and loses its load-bearing capacity.

### 2.3. Quasi-Brittle Peridynamics Model 

#### 2.3.1. Description of the Stretching Stage 

The mechanical behavior of quasi-brittle materials in the tensile phase, as described in Section 2.2, has been known to exhibit mainly brittle characteristics. When using PD to describe the behavior of brittle materials in the tensile phase, the bond forces are considered to be related only to the relative elongation of the bonds and can be described as:

(6) $$f\left(\eta ,\xi \right)=cs\left(\xi ,\eta \right)\frac{\xi +\eta }{\left|\xi +\eta \right|}$$

where c is the micro-elastic modulus, obtained from the consistency between the strain energy density of the isotropic material and the theoretical strain energy density of the continuum mechanics. The micro-elastic modulus of the isotropic material in the plane stress state is $c=9E/\left(\pi h\delta^{3}\right)$, where E is the elastic modulus and δ is the neighborhood radius.

#### 2.3.2. Description of the Compression Stage

In the compression stage, the quasi-brittle material can be divided into two phases: the elastic phase before reaching the elastic limit and the plastic phase after exceeding the elastic limit. For the elastic stage, the bond force is calculated in the same way as for the tensile stage. For the plastic stage, the quasi-brittle material exhibits plastic softening, and the influence of plastic deformation must be considered in the calculation of the bond force, which can be expressed as:

(7) $$f\left(\eta ,\xi \right)=c\left[s\left(\xi ,\eta \right)-s_{p}\left(\xi ,\eta \right)\right]\frac{\xi +\eta }{\left|\xi +\eta \right|}$$

where $s_{p}$ indicates the amount of plastic deformation after the bond enters the plastic phase. 

#### 2.3.3. Yield Criteria 

In compression, once the bond force exceeds the critical force of the elastic limit of the bond, plastic deformation occurs in the bond, resulting in the accumulation of damage. The bond force decreases due to the accumulation of plastic deformation, and the critical force of the bond decreases due to the accumulation of damage until the bond force is less than the critical force of the bond.

In order to accurately calculate the true bond force for quasi-brittle materials undergoing plastic softening behavior in the compression phase, the bond strengths need to be known. Chu et al. [18], in their work, expressed the bond strengths as follows by emulating the strength expressions in the JH-2 model in the classical framework [30]:

(8) $$p=p_{i}-D\left(p_{i}-p_{f}\right)$$

where $p_{i}$ is the critical force when the bond is undamaged, $p_{f}$ is the critical force when the bond is fully damaged, expressed as:

(9) $$p_{f}=\beta p_{i}$$

and D represents the cumulative damage from plastic deformation of the bond:

(10) $$D=\frac{\sum \Delta s_{p}}{s_{1}-s_{e}}$$

where $\Delta s_{p}$ is the plastic deformation in one time step, $\sum \Delta s_{p}$ is the accumulated plastic deformation, $s_{e}$ is the elastic compression limit, and $s_{1}$ is the plastic-compression deformation limit.

In order to characterize the relationship of the bond in the compressive plastic softening phase, it is necessary to define a function to determine whether the bond force has reached the maximum allowed value; referring to the theory of continuum-media mechanics, the yield criterion of the bond is expressed as:

(11) $$\varphi \left(s,s_{p},\dot{s}\right)=f\left(s\right)-p\left(s_{p},\dot{s}\right)=f-\left[1-\left(1-\beta \right)D\right]p_{i}$$

where $\dot{s}$ is the compressive deformation rate of the bond, which is the time derivative of the relative stretch of the bond, expressed as follows:

(12) $$\dot{s}\left(\xi ,\eta \right)=\frac{\;\dot{\eta }\left(\eta +\xi \right)}{\left|\eta \right|\left|\eta +\xi \right|}$$

#### 2.3.4. Flow Rule

Similar to the consistency condition in elastoplastic mechanics, the consistency condition for the bond is defined by deriving Equation (11) as follows:

(13) $$\dot{\varphi }\left(s,s_{p},\dot{s}\right)=\frac{\partial \varphi }{\partial f}\dot{f}-\frac{\partial \varphi }{\partial s_{p}}{\dot{s}}_{p}=\dot{f}+\frac{\left(1-\beta \right)p_{i}}{s_{1}-s_{e}}{\dot{s}}_{p}=0$$

where the second-order derivative of the relative deformation of the bond is neglected. According to Equation (13), the bond force remains equal to the elastic limit value of the bond. The derivation of Equation (7) is as follows:

(14) $$\dot{f}=c\left(\dot{s}-{\dot{s}}_{p}\right)$$

Based on Equations (13) and (14), the relative compression plastic deformation rate of the bond can be solved as:

(15) $${\dot{s}}_{p}=\frac{c}{c-H}\dot{s}$$

where $H=\frac{\left(1-\beta \right)p_{i}}{s_{1}-s_{e}}$.

#### 2.3.5. Consideration of Thermal Effects 

Since the mechanical behavior of the material in the elastic phase is not affected by the loading rate, the thermo-mechanical coupling bond-force function in this phase can be simply expressed in a linear form as follows:

(16) $$f\left(\eta ,\xi ,T\right)=c(T)\left[s-\Delta T_{avg}\alpha \left(T\right)\right]\frac{\xi +\eta }{\left|\xi +\eta \right|}$$

where α(T) is the coefficient of thermal expansion under different temperatures; and c(T) is the bond constant considering the effect of temperature, which is a material-dependent constant. This can be obtained by making the elastic strain energy density in the theory of elastic mechanics equal to the deformation energy density at the material point. The relation between c(T) and the elastic modulus of the material under a plane-stress state can be expressed as:

(17) $$c\left(T\right)=\frac{9E\left(T\right)}{\left(\pi h\delta^{3}\right)}$$

where the E(T) is Young’s modulus of the material at different temperatures.

When the bond is in the plastic phase, the bond-force function can be expressed as follows due to the plastic softening behavior and temperature effects:

(18) $$f\left(\xi ,\eta ,\dot{\eta },T\right)=c(T)\left[s\left(T\right)-s_{p}\left(\xi ,\eta ,\;\dot{\eta },T\right)\right]$$

Meanwhile, the yield function, Equation (13), which determines whether the bond force reaches the maximum permissible value, can be expressed as:

(19) $$\varphi \left(s,s_{p},\dot{s},T\right)=f\left(s,T\right)-p\left(s_{p},\dot{s},T\right)=f-\left[1-\left(1-\beta \right)D\left(T\right)\right]p_{i}$$

where D(T) is the damage that gradually accumulates, considering the thermal effect, could be expressed as:

(20) $$D(T)=D(T)+\frac{\sum \Delta s_{p}\left(T\right)}{s_{1}-s_{e}}$$

Here, $\Delta s_{p}\left(T\right)$ is the plastic compression deformation, considering the thermal effect, at each time step Δt, which can be expressed as:

(21) $$\Delta s_{p}\left(\xi ,\eta ,\;\dot{\eta },T\right)=\dot{s}\left(\xi ,\eta ,\;\dot{\eta },T\right)\Delta t-\frac{\Delta f}{c(T)}$$

To obtain the change rate of bond stretching $\dot{s}\left(\xi ,\eta ,\;\dot{\eta },T\right)$, we derived Equation (12):

(22) $$\dot{s}\left(\xi ,\eta ,\;\dot{\eta },T\right)=\frac{\;\dot{\eta }\left(\eta +\xi \right)}{\left|\eta \right|\left|\eta +\xi \right|}-\frac{\Delta T_{avg}}{\Delta t}\alpha$$

It should be noted that the consistency condition also needs to be satisfied, i.e., the time derivative of Equation (13):

(23) $$\dot{\varphi }\left(s,s_{p},\dot{s},T\right)=\frac{\partial \varphi }{\partial f}\dot{f}-\frac{\partial \varphi }{\partial s_{p}}{\dot{s}}_{p}=\dot{f}\left(s,T\right)+\frac{\left(1-\beta \right)p_{i}}{s_{1}-s_{e}}{\dot{s}}_{p}\left(s,\dot{s},T\right)=0$$

The relationship between $\dot{s}\left(\xi ,\eta ,\;\dot{\eta },T\right)$ and ${\dot{s}}_{p}\left(\xi ,\eta ,\;\dot{\eta },T\right)$ can be similarly obtained from the above equation and Equation (15) and is expressed as:

(24) $${\dot{s}}_{p}\left(\xi ,\eta ,\;\dot{\eta },T\right)=\frac{c\left(T\right)}{c\left(T\right)-H}\dot{s}\left(\xi ,\eta ,\;\dot{\eta },T\right)$$

Therefore, the BB-PD thermo-mechanical coupling equations applicable to quasi-brittle materials are as follows:

(25) $$\left\{ \begin{array}{l} \rho c_{v}\dot{T}\left(x,t\right)=\int_{H}\left(\kappa \frac{\tau }{\left|\xi \right|}-T_{0}\frac{c\alpha }{2}\dot{e}\right)+h_{s}\left(x,t\right)\\ \rho \;\ddot{u}\left(x,t\right)=\int_{H}\frac{\xi +\eta }{\left|\xi +\eta \right|}f\left(\xi ,\eta ,T\right)dV_{\;x^{\prime }}+b\left(x,t\right) \end{array}\right.$$

### 2.4. Numerical Discretization and Time Integration

For Equation (25), the motion equation and heat conduction can be replaced by a discretized form, as given below:

(26) $$\left\{ \begin{array}{l} \rho_{i}c_{i}{\dot{T}}_{i}\left(t\right)=\sum_{j=1}^{N_{i}}\mu_{ij}\left(h_{cij}\left(t\right)\kappa_{ij}\frac{T_{i}-T_{j}}{\left|\xi_{ij}\right|}-T_{i,0}\frac{c_{ij}\alpha_{ij}}{2}{\dot{e}}_{ij}\right)V_{j}+h_{s,i}\left(t\right)\\ \rho_{i}{\ddot{u}}_{i}\left(t\right)=\sum_{j=1}^{N_{i}}f_{ij}\frac{\xi_{ij}+\eta_{ij}}{\left|\xi_{ij}+\eta_{ij}\right|}V_{j}+b_{i}\left(t\right) \end{array}\right.$$

where $f_{ij}$ is the bond force function between the matter points i and j. This can be calculated using Equation (16) in the elastic phase and Equation (18) in the plastic phase.

In this study, in order to describe the thermo-mechanical coupling in the framework of the PD model of quasi-brittle materials, an interleaved scheme is used to approximate the solution. This means that the coupled system equations are solved separately, and different time-explicit algorithms are employed to solve the heat conduction equation and the kinetic equation. In particular, the explicit integration algorithm with the first-order forward difference is used to solve the heat conduction equation, and the temperature of the next time step is obtained as:

(27) $$T_{\left(i\right)}\left(t+\Delta t\right)=T_{\left(i\right)}\left(t\right)+\Delta t^{T}\cdot {\dot{T}}_{\left(i\right)}\left(t\right)$$

where $\Delta t^{T}$ is the thermo-mechanical time step.

In addition, similar to the quasi-static problem, virtual inertia and damping terms are introduced to solve the dynamics equations, which can be expressed as:

(28) $$D\ddot{u}\left(x,t\right)+cD\dot{u}\left(x,t\right)=f\left(u_{i},u_{j},x_{i},x_{j}\right)$$

where *D* is the virtual diagonal density matrix and *c* is the damping coefficient, obtained from Reschgorin law and Rayleigh quotient [31], respectively. Using the central difference algorithm, the displacement and velocity for the next time step are defined as:

(29) $${\dot{u}}^{n+1/2}=\frac{\left(2-c_{n}\Delta t\right){\dot{u}}^{n-1/2}+2\Delta t^{M}D^{-1}F^{n}}{\left(2+c^{n}\Delta t^{M}\right)}$$

(30) $$u^{n+1}=u^{n}+\Delta t^{M}\cdot {\dot{u}}^{n+1/2}$$

where $\Delta t^{M}$ is the kinetic time step.

In thermo-mechanical coupled problems, the kinetic characteristic time scale depends on the propagation velocity of the stress wave in the material, and the heat conduction characteristic time scale depends on the thermal diffusivity of the material. In general media, the time scale of heat transfer characteristics is usually much larger than that of kinetic characteristics. Therefore, the whole thermal coupling solution can be divided into the following three steps.

Step 1: The heat conduction equation is solved and the temperature field distribution of the whole model is calculated.

Step 2: The motion equations are solved until the whole model reaches a steady state.

Step 3: The heat conduction equation is solved for the next thermo-mechanical time step.

The above steps are repeated to obtain the entire thermo-mechanical coupling solution. It should be noted that the convergence criterion is also needed to determine the steady state of the kinetic iterative solution. When the whole model reaches the steady state, the displacement increment of each kinetic iteration step tends to 0. Chen et al. [14] have previously defined the parametric number, Re, as shown in Equation (32), and provided a minimal value, Ω. When Re≤Ω, the system reaches the steady state and can be solved in the next thermo-mechanical time step. Otherwise, the iteration continues until it converges with the formula below:

(31) Re=∑m=1M(umi,j−umi,j−1)2M

where M is the entire number of model particles.

## 3. Model Verification and Convergence Analysis

In this section, the BB-PD model proposed in Section 2 is implemented in Fortran code, and two typical cases are applied to verify the efficiency of the model. The convergence of the numerical model is also analyzed in the following section.

### 3.1. Ceramic Plates Subjected to Heating Loads

**Figure 4:**
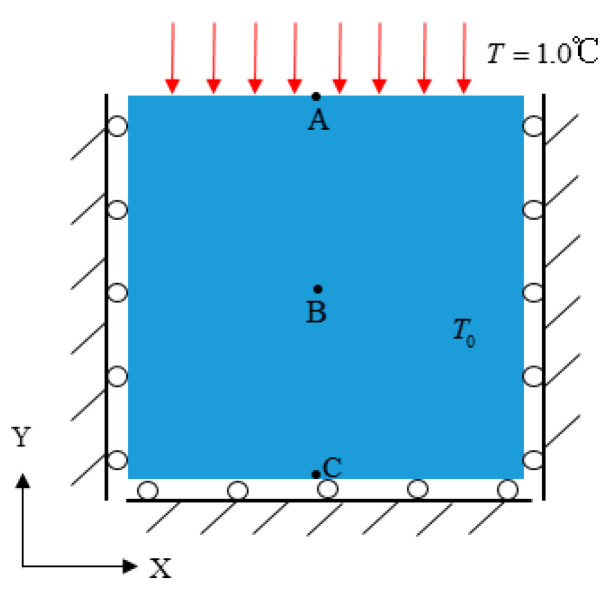
The two-dimensional flat plate subjected to heating loading.

Figure 4 shows the computational model of the ceramic plates subjected to heating loads. A flat directional plate with a side length *L* = *W* = 1 m is adiabatically constrained to the normal directional displacement with respect to three sides, except for the top. The initial temperature of the whole plate is 0 °C and the temperature of *T* = 1.0 °C is applied to the top boundary. The peridynamic, mechanical, and thermo-mechanical parameters used in the numerical simulation are listed in Table 1. Three points, referred to as A, B, and C on the vertical symmetry axis at the center of the plate, and located on the top boundary, positive center, and bottom boundary of the plate, respectively, were selected as reference points.

An identical finite element model with the same material properties was built using the commercial ABAQUS finite element software. The model was discreted into 200×200 grids with a time step of $1\times 10^{-5}\;s$, and a direct thermo-mechanical coupling method was used. Additionally, the theoretical calculation formulas for the temperature and vertical displacement of three reference points were provided by Timoshenko [32] and Carslaw [33]:

(32) $$T\left(y,t\right)=1-\frac{4}{\pi }\sum_{n=0}^{\infty }\frac{{\left(-1\right)}^{n}}{2n+1}\exp\left(-\frac{\left(2n+1\right)\pi^{2}kt}{4L^{2}}\right)\cos\left(\frac{\left(2n+1\right)\pi y}{2L}\right)$$

(33) $$u_{y}\left(y,t\right)=\left(1+\nu \right)\alpha \int_{0}^{y}T\left(y,t\right)dy$$

**Figure 5:**
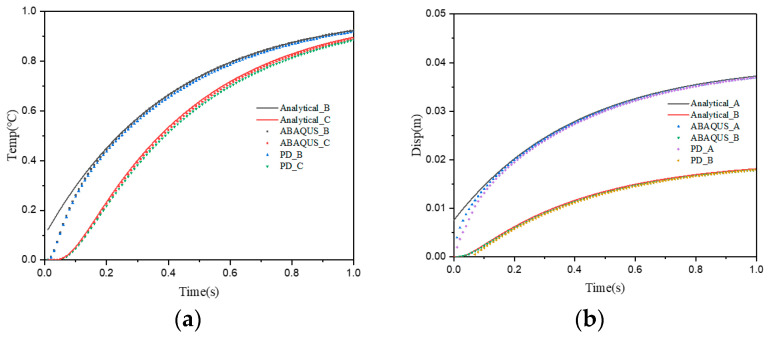
Comparison of calculation results of different methods. (**a**) Temperature; (**b**) Vertical displacement.

In both simulations, in spite of the temperature loads applied, the temperature change causes deformation inside the plate due to the thermo-mechanical coupling effect, and the reference points are displaced at the same time. The simulation results of different reference points according to both models are shown in Figure 5, the calculation results of PD and ABAQUS are consistent with those of analytical results, thus, demonstrating the reliability of the proposed approach in solving the thermo-mechanical coupling problem.

### 3.2. Pre-Cracked Brazilian Disk under Uniaxial Compression

**Figure 6:**
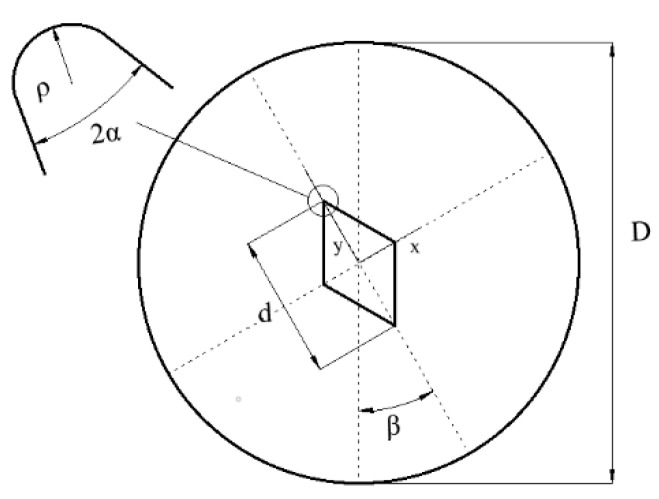
The VBD specimen used in experiments.

The Ayatollahi’s experiments on the brittle fracture of polycrystalline graphite were simulated [34]. A modified version of the cracked Brazilian disk (CBD) specimen called the V-notched Brazilian disk (VBD) specimen was used in this experiment. As shown in Figure 6, the specimen is a circular disk of diameter D containing a central rhombic hole with an opening angle 2α and length d for the VBD specimen. The disk diameter and the notch depth were 60 mm and 15 mm, respectively, and the angles used in the experiment were 2α=30°, β=15°. The basic material properties of polycrystalline graphite are as follows: density of $1710\;\mathrm{kg}/m^{3}$, Young’s modulus of 8.05 GPa, Poisson’s ratio of 0.33, and the fracture toughness of $1.0\;\mathrm{MPa}{\;m}^{0.5}$. In the experiment, the fracture test was performed by using a universal tension–compression test machine under displacement conditions with a loading rate of 0.05 mm/min.

**Figure 7:**
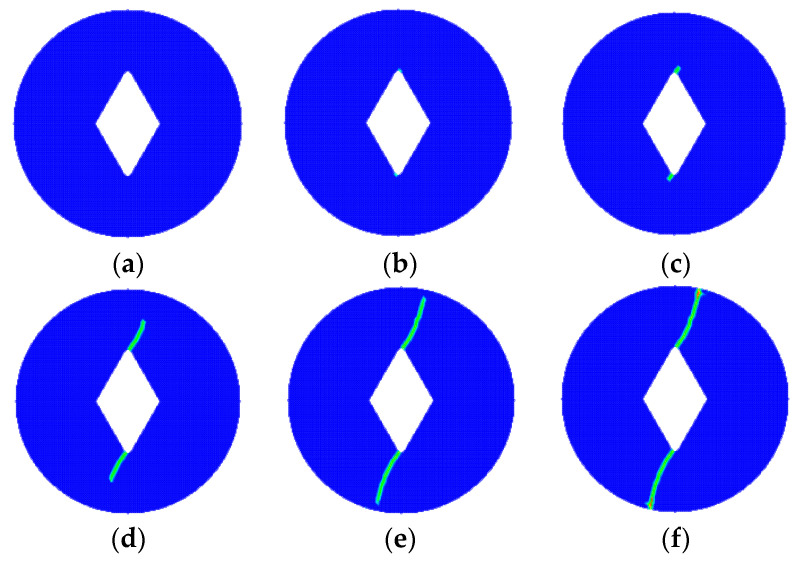
Splitting and destruction process of the VBD specimen. (**a**) 0 s; (**b**) 70 s; (**c**) 80 s; (**d**) 110 s; (**e**) 130 s; (**f**) 170 s.

Using the proposed model to simulate the crack extension of the VDB specimen, the splitting damage process is shown in Figure 7. At 70 s, the tips of both sides of the pre-existing crack begin to accumulate damage due to stress concentration exceeding the strength limit, and crack initiation occurs here. Then at 80 s, cracks appear at the tips of both sides of the pre-existing crack and begin to propagate outward. Next, at 110 s, the cracks on both sides remain symmetrical and propagate to both sides of the loading. Finally, at 170 s, cracks penetrate the tips of both ends of the precast crack and both sides of the loading point, at which point the Brazilian disc specimen fails.

**Figure 8:**
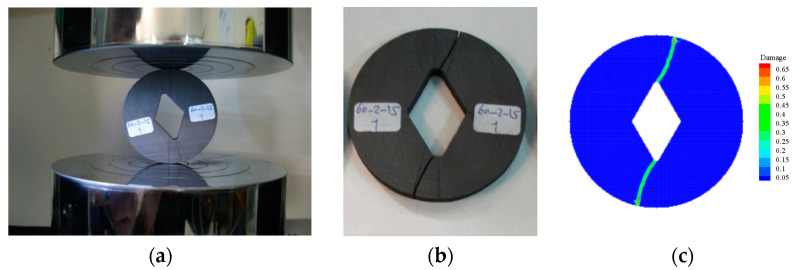
(**a**) Specimen before experiment; (**b**) Specimen after experiment; (**c**) The PD simulation result.

The comparison of the prefabricated cracked Brazilian disc splitting damage before and after the experiment with the PD simulation results is shown in Figure 8. It can be observed that the simulated results are in high agreement with the experimental results.

### 3.3. Convergence Analysis

Furthermore, the numerical convergence of the model was also analyzed using the case study of Section 3.1. Currently, the convergence analysis for PD consists of two main types: *m*-convergence and δ-convergence [35]. 

**Figure 9:**
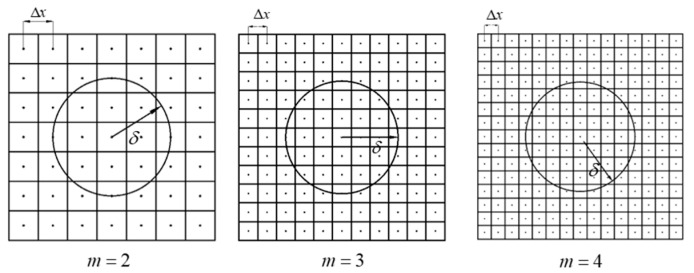
*m*-convergence with a fixed horizon size.

**Figure 10:**
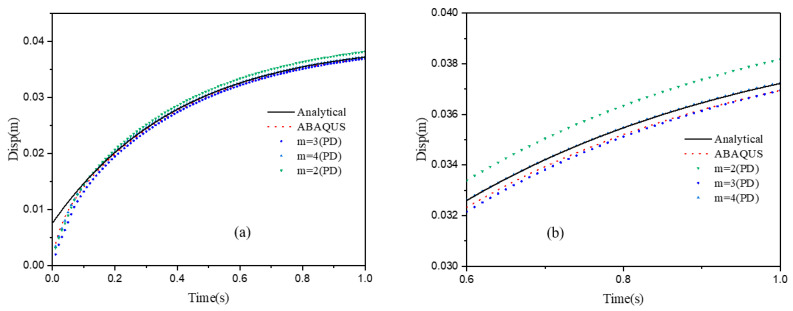
(**a**) The vertical displacement of point A with a different non-locality parameter *m*; (**b**) an enlarged detail from (**a**).

In the *m*-convergence, horizon δ is kept constant as $\delta =5.0\times 10^{-3}\;m$ throughout the computation, while *m* and Δx are taken as 2, 3, and 4, and $2.5\times 10^{-3}\;m$, $1.66\times 10^{-3}\;m$, and $1.25\times 10^{-3}\;m$, respectively, as shown in Figure 9. The vertical displacements of the reference point obtained usinig the proposed model, the analytical solution, and the FEM simulation are shown in Figure 10. For a fixed horizon, as the value of m increases, the error rate between the simulation results and the analytical results becomes smaller. Although the error can be captured when m=3 or 4, m=3 is preferred, considering the effect of computational efficiency.

**Figure 11:**
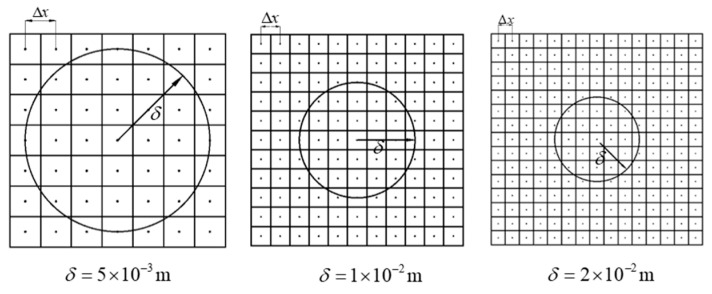
δ-convergence with a fixed parameter *m*.

**Figure 12:**
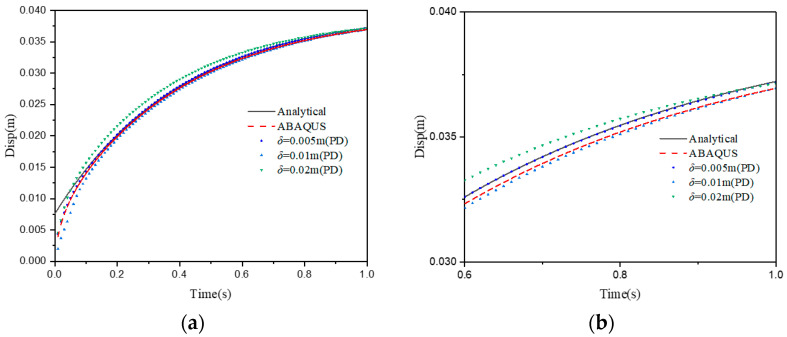
(**a**) The vertical displacement of point A with different horizon sizes δ; (**b**) an enlarged detail from (**a**).

In the δ-convergence, the non-locality parameter *m* is kept as a constant, i.e., *m* = 3. Three different horizon sizes are chosen as $\delta =1.5\times 10^{-2}\;m$, $3\times 10^{-2}\;m$, $6\times 10^{-2}\;m$, and $\Delta x=5\times 10^{-3}\;m$, $1\times 10^{-2}\;m$, $2\times 10^{-2}\;m$ (see Figure 11). The vertical displacements calculated at reference points using the different δ are compared to those obtained from the finite element method, as shown in Figure 12. For a fixed non-locality parameter, with a decrease in the horizon sizes, the error calculation will also decrease, but it will lead to an increase in the calculation efficiency. Therefore, choosing the right value of horizon sizes requires special consideration.

## 4. Numerical Applications

The previous studies have shown that the proposed model is able to accurately simulate the mechanical behavior of quasi-brittle materials under thermal loading (in Section 3.1) and static loading (in Section 3.2). For the purpose of clarifying the applicability of the model, this section applies it to two more complex coupled thermal-force processes: the ceramic quenching process (in Section 4.1) and the process of compressing the rock after heat treatment (in Section 4.2).

### 4.1. Ceramic under Cold Shock

**Figure 13:**
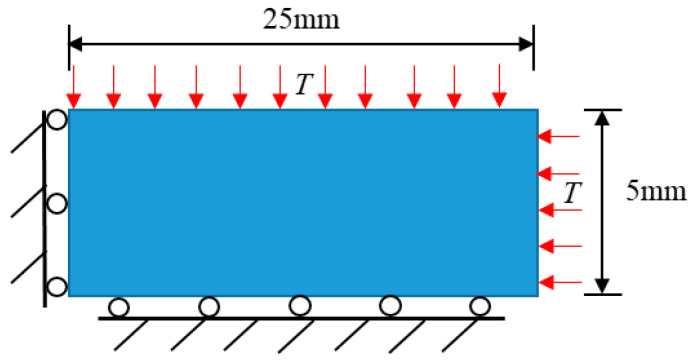
Schematic diagram of the geometry and boundary condition of the ceramic subjected to cold shock.

Referring to the quenching experiments of ceramic plates at different temperatures conducted by Jiang et al. [36], an alumina ceramic plate with the dimensions of 50 mm×10 mm is heated to 873 K and subsequently allowed to freefall into the water at 293 K. Considering the symmetry of the load and boundary conditions, a 1/4 model, shown in Figure 13, could be established to perform the calculation. In such a model, the left and lower boundaries are constrained during the normal displacements, and a uniform and constant-cold impact load is applied to the upper and right boundaries. The convective heat transfer coefficient $h=70,000\;W/\left(m^{2}\cdot K\right)$ is taken when the ceramic plate is dropped into the water. The PD and thermo-mechanical parameters involved in the numerical simulations are listed in Table 2.

When the high-temperature ceramic plate (873 K) enters the room-temperature water (293 K), the heat energy of the plate spreads rapidly to the surrounding ambient medium. The surface temperature of the ceramic plate decreases sharply, as shown in Table 3, forming a vast temperature gradient with the interior. This temperature gradient from the inside to the outside (i.e., hot inside and cold outside) causes the ceramic surface to undergo tensile stress while the interior is subjected to compressive stress. When the tensile stress on the ceramic surface exceeds that of the interior of the ceramics, damage occurs, and cracks propagate throughout the material. Since the temperature bond also breaks due to the thermal effect, heat conduction through the crack is blocked and the temperature on both sides of the bending crack exhibits a significant temperature jump.

For cracks due to thermal shock, all cracks are distributed in a parallel manner at equal distances on the outer surface of the ceramic (upper and right side) and extend from the outside to the inside. As the cold shock continues, some of the initial cold shock cracks stop growing, while other thermal shock cracks continue to grow.

During the first 10 ms, the cracks are uniformly distributed at intervals of roughly 0.001 mm, and the length of each crack remains consistent. As the cold shock continues, some initial cold shock cracks stop growing at 50 ms, while other heat shock cracks continue to grow. Thereafter, the ceramic plate temperature gradient decreases, the thermal stress becomes smaller, the crack expansion slows down, and the crack stops growing at 600 ms, reaching the maximum length.

**Figure 14:**
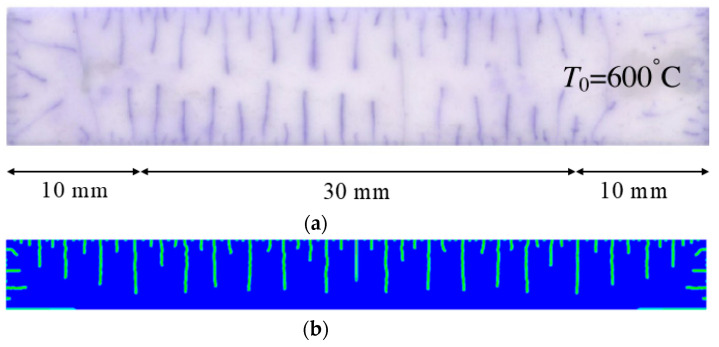
Comparison of ceramic plate thermal impact cracking results: (**a**) Specimens after thermal shock [36]; (**b**) PD simulation results for the 1/2 model.

In the work of Jiang et al. [36], the area within 10 mm of the ends of the specimen was excluded in order to eliminate the effect of the end boundaries. The average dimensionless crack spacing $\bar{s}$ and dimensionless crack length $\bar{p}$ were proposed, denoted as $\bar{s}=s/L_{C}$ and $\bar{p}=p/L_{C}$, respectively, where s is the crack spacing, p is the crack length, and $L_{C}$ is the specimen width. The average dimensionless crack spacing in the simulation results is 0.112, compared with 0.12 in experiments, and the dimensionless crack length in the simulation results is 0.715, compared with 0.79 in experiments [36]. The thermal impact cracks show a clear spacing distribution, i.e., there are short cracks in the middle of long cracks. The comparison between the simulated and experimental results is shown in Figure 14, where the thermal impact cracks remain similar in terms of spacing, length, length hierarchy, and periodicity. However, since the model used in the experiments is not an ideal model, the ceramic plate is a non-homogeneous material and there are small gaps in the structure, which cannot be consistent with the simulated results, as evidenced by the asymmetry of the thermal cracks in the experimental results.

### 4.2. Granite under Uniaxial Compression after Heat Treatment

**Figure 15:**
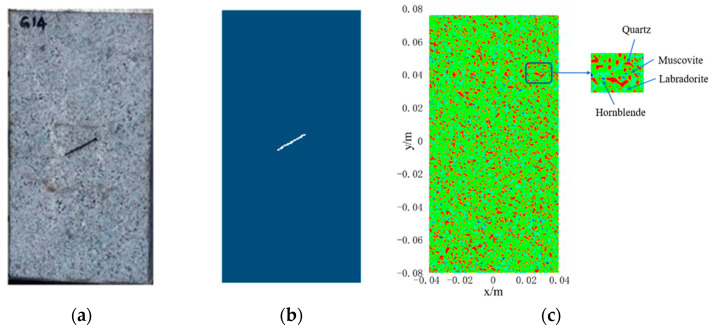
(**a**) Granite samples containing prefabricated cracks; (**b**) PD model; (**c**) composition distribution of granite.

The granite specimen with prefabricated cracks was first subjected to thermocycling and then compressed uniaxially, as performed by Yang et al. [37]. The dimensions of the granite specimen were 80 mm×160 mm (see Figure 15a), and there was a prefabricated crack with a length of 20 mm, width of 1.5 mm, and inclination angle of 30° in the center of the specimen. At the thermal cycling stage, Yang et al. first heated the granite specimen to 573 K and then kept the temperature constant to make the inside and outside of the sample converge to the same temperature. Subsequently, the sample was placed in the open air and cooled down naturally to room temperature (293 K). At the uniaxial compression stage, the top and bottom ends of the specimens were loaded in compression using a loading speed of 0.1m/s, and the crack nucleation and expansion were observed. The mechanical and thermo-mechanical parameters in the PD simulation are listed in Table 4.

Before the numerical simulation, the same geometric model as that for the granite specimen was established, as shown in Figure 15b. Due to the non-homogeneous properties of rock materials, the Weibull distribution is often introduced to describe the statistical distribution of the characteristic parameters [38], such as elastic modulus, Poisson’s ratio, and thermal expansion coefficient in the PD simulation. However, the Weibull distribution does not accurately reflect the properties of granite due to the variety of mineral components and contents of rocks and their vastly different material properties. Therefore, this study consists of reconstructing the PD calculation model of the non-homogeneous granite with the non-uniform and discontinuous thermal expansion coefficients using the Knuth–Durstenfeld stochastic algorithm proposed by Yang et al. [17] (see Figure 15c). The proportions of mineral compositions and thermal expansion coefficients of granite materials are listed in Table 5.

**Figure 16:**
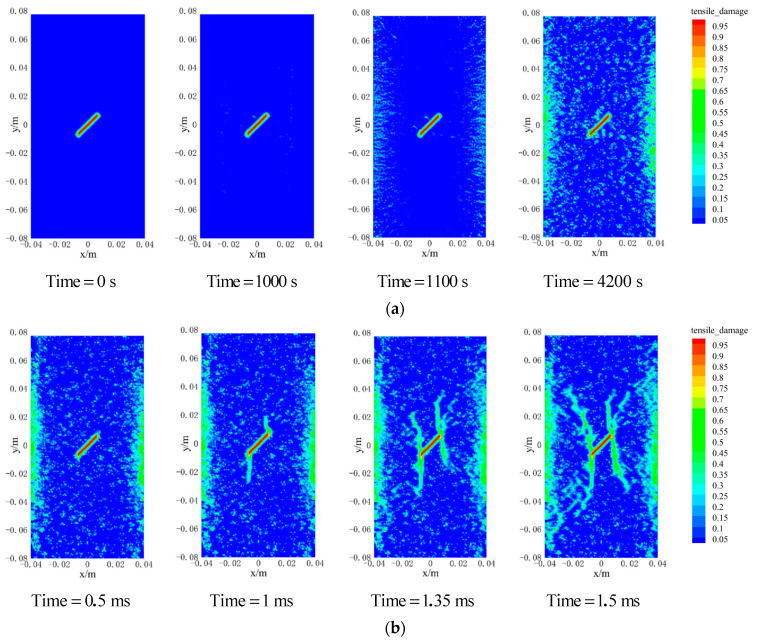
PD simulation of damage in granite under uniaxial compression after thermal cycling. (**a**) Thermal cycle stage; (**b**) Uniaxial compression stage.

After 1000 s of thermal loading, the temperature of the granite specimen increased from 293 K to 573 K. Subsequently, after 3200 s, the sample naturally cooled down to room temperature (293 K). The simulation of the whole heat treatment process is shown in Figure 16a. Due to the slow temperature rise at the warming stage, the temperature difference between the inside and outside of the granite is small, and the non-uniform thermal stress caused by the temperature gradient is minor. Therefore, only a tiny amount of discontinuous thermal cracks is generated inside the granite during the entire heating-up stage. In addition, the higher compression strength of the granite also suppresses the crack generation at the warming stage. Unlike the warming stage, the outer surface of the granite decays sharply to room temperature during the natural cooling stage. This drastic heat transfer behavior leads to the formation of a vast temperature gradient inside and outside the granite, which provokes a rapid increase of the tensile (thermal) stress on the surface of the specimen under tensile strength, causing more discontinuous cracks to occur on both sides of the sample. These cracks continue to expand in the course of the cooling process and then gradually penetrate and fall off. Moreover, due to the inconsistency between the thermal expansion coefficients of different mineral components inside the specimen, there are more and more cracks induced by uneven thermal expansion.

The crack initiation and propagation process of the granite specimens containing prefabricated cracks under uniaxial compression simulated through the PD model is shown in Figure 16b. In a future study, the crack types will be analyzed according to the classification of crack types proposed by Yang et al. [38]. At the initial stage of loading, the main strain concentrations are found from the tips of the pre-existing fissure; the granite specimens had no macroscopic crack generation except for a large number of thermal micro-cracks on both sides, due to the thermal cycling process. With the increase of load, when the time reached 1 ms, the main strain concentrations develop obviously, the secondary tensile crack appeared at both the upper and lower ends of the prefabricated crack of the specimen, but the development of the secondary tensile crack was not symmetrical due to the uneven distribution of thermal micro-cracks inside the model. Subsequently, at 1.35 ms, a downward expanding tensile wing crack appeared at the upper end of the precast crack, while an upward expanding anti-shear crack appeared at the lower end of the precast crack. At the same time, thermal micro-cracks can be observed developing into secondary tensile cracks on both sides of the prefabricated cracks that afterward form web-like cracks. The macroscopic cracks in the final granite specimens were classified as secondary tensile cracks and anti-shear cracks.

**Figure 17:**
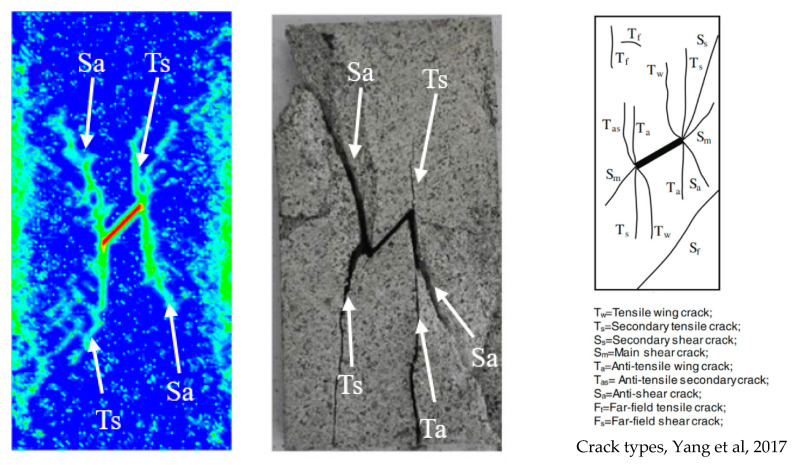
Comparison of PD simulated cracks extension with experiment [37,38].

The comparison between the experimental and PD simulation results of uniaxial compression after thermal cycling of precast cracked granite is shown in Figure 17. Secondary tensile cracks and anti-shear cracks existed at the tips of both sides of the precast cracks and were approximately the same in the form of extension. However, both tensile wing cracks and anti-shear cracks exist on the right side of the precast crack in the experimental results [37], while only anti-shear cracks exist in the simulation process. This is caused by the existence of tiny voids inside the granite, which is a non-homogeneous material, and the presence of a large number of non-uniformly distributed thermal micro-cracks during the thermal cycling process, which prevent the anti-tensile cracks that should appear along the axial stress direction; hence, only the anti- shear cracks mainly caused by shear damage appeared. The inconsistency of crack forms on both sides in the experimental results also indicates the non-homogeneous nature of granite.

Existing studies have shown that the deformation process of concrete is very complex due to its heterogeneity and involves progressive damage, such as the generation, propagation, and coalescence of microcracks [39]. While we have considered the parameters of the different components of the granite in an effort to construct a macroscopic heterogeneous model subjected to thermo-mechanical coupling loads, we neglected the mechanical characteristics at the microscopic scale. Through the references [40,41,42,43,44], it may be observed that the study of thermo-mechanical coupling under microstructures tends to focus on the mechanical properties of microstructures using the theory of nonlocal elasticity, by combining intermolecular or interatomic bonds into their specific intrinsic structural relationships. Further study in this area would increase the validity of the model.

## 5. Conclusions

In this paper, a coupled model capable of simulating the thermal-force damage behavior of quasi-brittle materials was proposed based on the bond-based PD theory using the fully thermodynamic coupling equation. The model consists in describing different mechanical properties of quasi-brittle materials in tensile and compressive states, constructing bond force functions in the tensile and compressive phases, and introducing the role of temperature terms in the bond-based peridynamic model. The simulations of the thermal expansion process of ceramics and the static compression damage of polycrystalline graphite were applied to the proposed model, and the numerical model results showed agreement with the experimental results in the references. In addition, the model was also used to simulate thermal damage processes in ceramics and in homogeneous rocks, revealing the potential capacity of the model in analyzing the post-thermal damage behavior of quasi-brittle materials.

## Figures and Tables

**Table 1:** Parameters involved in the numerical simulation.

	Parameter	Value
PD parameters	Number of discrete points in the xy direction	200 × 200
	Material point spacing Δx (m)	0.005
	non-locality parameter m	3
Mechanical parameters	Heat transfer time step $\Delta t^{TH}$ (s)	$1\times 10^{-5}$
	Young’s modulus E (GPa)	1
	Poisson’s ratio ν	0.33
	Density ρ ($\mathrm{kg}/m^{3}$)	1
Thermal parameters	Thermal conductivity $k_{T}$ ($W\cdot m^{-1}K^{-1}$)	1
	Coefficient of thermal expansion α (1/K)	0.02
	Specific heat capacity $c_{v}$ ($J\cdot {\mathrm{Kg}}^{-1}K^{-1}$)	1

**Table 2:** Parameters involved in the PD model of the ceramic under cold shock.

	Parameter	Value
PD parameters	Number of discrete points in the xy direction	500 × 100
	Material point spacing Δx (m)	0.00005
	Non-locality parameter m	3
Mechanical parameters [36]	Heat transfer time step $\Delta t^{TH}$ (s)	$1\times 10^{-4}$
	Young’s modulus E (GPa)	370
	Poisson’s ratio ν	0.33
	Density ρ ($\mathrm{kg}/m^{3}$)	3980
	Fracture energy $G_{0}$ ($J/m^{2}$)	24.3
Thermal parameters [36]	Thermal conductivity $k_{T}$ ($W\cdot m^{-1}K^{-1}$)	31
	Coefficient of thermal expansion α (1/K)	$7.5\times 10^{-6}$
	Specific heat capacity $c_{v}$ ($J\cdot {\mathrm{Kg}}^{-1}K^{-1}$)	880

**Table 3:** The temperature field and crack evolution in high-temperature ceramics under cold shock loading.

	Temperature Field (K)	Evolution of Cracks (Damge)
Time = 10 ms	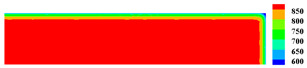	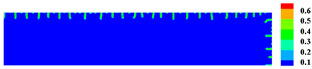
Time = 50 ms	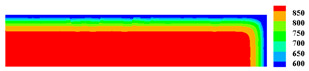	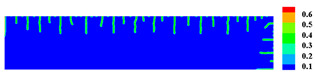
Time = 100 ms	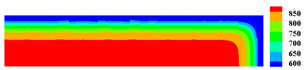	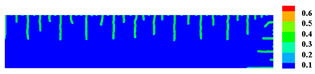
Time = 300 ms	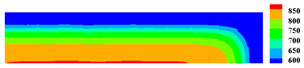	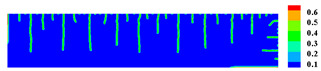
Time = 600 ms	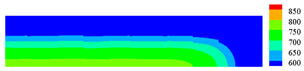	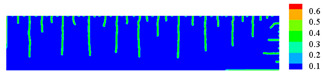

**Table 4:** Peridynamic, mechanical, and thermal parameters of the PD numerical model.

	Parameter	Value
PD parameters	Number of discrete points in the xy direction	100 × 200
	Material point spacing Δx (m)	0.00008
	Non-locality parameter m	3
Mechanical parameters [37]	Heat transfer time step $\Delta t^{TH}$ (s)	$2\times 10^{-4}$
	Mechanical time step during single-axis compression $\Delta t^{ME}$ (s)	$5\times 10^{-8}$
	Young’s modulus E (GPa)	36
	Poisson’s ratio ν	0.33
	Density ρ ($\mathrm{kg}/m^{3}$)	2790
	Fracture energy $G_{0}$ ($J/m^{2}$)	50
Thermal parameters [37]	Thermal conductivity $k_{T}$ ($W\cdot m^{-1}K^{-1}$)	3.5
	Specific heat capacity $c_{v}$ ($J\cdot {\mathrm{Kg}}^{-1}K^{-1}$)	900

**Table 5:** Proportions of mineral compositions and thermal expansion coefficients of granite materials.

Type of Mineral 1	Proportion (%)	Coefficient of Thermal Expansion $\left(10^{-6}K^{-1}\right)$
Quartz	17.73	24.3
Muscovite	36.33	17.3
Labradorite	39.32	14.1
Hornblende (rock-forming mineral, type of amphibole)	6.62	8.7

## Data Availability

The data used to support the findings of this study are available upon request from the corresponding author.
